# Comparison of Older and Newer Generation Active Style Pro Accelerometers in Physical Activity and Sedentary Behavior Surveillance under a Free-Living Environment

**DOI:** 10.3390/ijerph16091597

**Published:** 2019-05-07

**Authors:** Shohei Yano, Mohammad Javad Koohsari, Ai Shibata, Kaori Ishii, Levi Frehlich, Gavin R. McCormack, Koichiro Oka

**Affiliations:** 1 Institute for Sport Sciences, Waseda University, Tokorozawa 359-1192, Japan; 2 Faculty of Sport Sciences, Waseda University, Tokorozawa 359-1192, Japan; Javad.Koohsari@baker.edu.au (M.J.K.); ishiikaori@waseda.jp (K.I.); koka@waseda.jp (K.O.); 3 Behavioural Epidemiology Laboratory, Baker Heart and Diabetes Institute, Melbourne 3004, Australia; 4 Faculty Health and Sports Sciences, University of Tsukuba, Tsukuba 305-8574, Japan; shibata.ai.ga@u.tsukuba.ac.jp; 5 Department of Community Health Sciences, Cumming School of Medicine, University of Calgary, Calgary, AB T2N 4Z6, Canada; lcfrehli@ucalgary.ca (L.F.); Gavin.McCormack@ucalgary.ca (G.R.M.)

**Keywords:** monitoring, objective assessment, accelerometry, motion sensors

## Abstract

*Background*. Comparability of accelerometers in epidemiological studies is important for public health researchers. This study aimed to compare physical activity (light, LPA; moderate, MPA; and moderate-to-vigorous, MVPA) and sedentary behavior (SB) data collected using two Omron triaxial accelerometer generations (Active style Pro, ASP) among a sample of Japanese workers in a free-living environment. *Methods*. Thirty active and sedentary workers (24–62 years) wore two types of ASP accelerometers, the HJA-350IT (350IT) and the HJA-750C (750C), simultaneously for seven consecutive days to represent a typical week. The accelerometers estimated daily average step counts and time spent per day in LPA, MPA, and MVPA. If a participant had data for ≥4 days (>10 h/day) it was considered valid. The difference and agreement between the two ASPs were analyzed using a paired *t*-test, intra-class correlation coefficients (ICC), and a Bland–Altman analysis in total and for each type of worker. *Results.* Among all workers, the 750C measured significantly (*p* < 0.05) less SB, MPA, MVPA, and more LPA compared with the 350IT. The agreements in ICC were high (ICC ≥ 0.94). *Conclusions.* Compared with the 350IT, the newer generation 750C ASP accelerometer may not provide equivalent estimates of activity time, regardless of the type of physical activity.

## 1. Introduction

Numerous studies have demonstrated the health benefits of engaging in moderate-to-vigorous physical activity (MVPA) such as walking, jogging, and exercise [1,2,3]. Recent evidence has also shown the negative health effects of sedentary behavior (SB) and in particular, spending too much time sitting [4,5]. Accurate and sustainable measurement of physical activity and SB is important to accumulate evidence for public health professionals. Physical activity measurement methods can be broadly classified as self-report and objective approaches [6,7]. In particular, accelerometers, because of their high reliability and validity, are frequently used to objectively measure active and sedentary behaviors under laboratory and free-living environments [8,9]. Moreover, recently accelerometers have been used in large epidemiological studies and longitudinal studies to measure physical activity and SB [10,11,12].

Over the past decade, several accelerometers have been employed to measure physical activity and SB [8,13]. One frequently used research-grade triaxial accelerometer is the Omron Active style Pro (ASP) HJA-350IT (350IT) (Omron Healthcare, Kyoto, Japan). Compared with the Douglas bag method for estimating representative activities of daily life (i.e., sedentary, household, and locomotive activities), the 350IT has a high validity especially for measuring light physical activity (LPA) [14]. In addition, the 350IT has shown a high correlation (r = 0.84) with the ActiGraph GT3X+ accelerometer (ActiGraph, Pensacola, FL, USA) in measuring SB [15]. The 350IT has been used in several epidemiological studies since its development in 2008 [16,17,18]. For example, a population-based study conducted in Japan found a positive association between moderate physical activity (MPA) measured by the 350IT and cognitive function among older adults [17]. Elsewhere, a study using the 350IT found substituting small amounts of SB and LPA with MVPA to be associated with better physical function in an elderly population [18]. In another study, the 350IT-measured LPA was associated with metabolic syndrome, independent of MVPA [19].

Omron Healthcare recently developed a new version of the ASP triaxial accelerometer, named the HJA-750C (750C), to replace the old generation 350IT, which is no longer commercially available. Compared with the older device (350IT), the new device (750C) is smaller, lighter, and can be worn on the wrist, which could improve participant accelerometer wear time—a major limitation of accelerometer data collection in large-scale surveys [20].

While the majority of the research to date has employed the 350IT, the 350IT and the 750C have the same proprietary algorithm for estimating physical activity. However, it is still important to examine whether there are any disagreements between the two devices in assessing time spent in different intensities of physical activity. As the algorithm has not changed between the two devices, it can be hypothesized that there should be no significant differences in measuring physical activity and SB between the two under a free-living environment. However, if differences do exist between the 350IT and the 750C, the 750C can’t be used interchangeably and compared with previous studies using the 350IT for measuring physical activity and SB. Additionally, if differences do exist, validity and calibration studies would be needed for the 750C. Moreover, if total physical activity is not different between the two devices, they still may differ in the type of physical activity measured (i.e., LPA, MPA, MVPA). The type of physical activity is an important factor affecting accelerometer measurement properties [21].

To our knowledge, there are currently no studies that compare the 350IT with the 750C for measuring physical activity and SB, conducted in an uncontrolled or free-living environment. Therefore, the purpose of this study was (1) to determine whether activity and SB measured by the new generation 750C device is comparable with that of the old generation 350IT device, among a sample of Japanese workers in a free-living environment, and (2) to confirm whether the differences (if any) are affected by the type of occupational physical activity (active vs. sedentary).

## 2. Materials and Methods

### 2.1. Participants

A convenience sample of 30 participants was recruited via word-of-mouth at a hospital and a university in Tokyo, Japan. Inclusion criteria included being able to undertake normal activities of daily living during the week of monitoring and walk without help or support. The participants were full-time workers aged 24 to 62 years. Our sample size of 30 was sufficient to detect an effect size of *d* = 0.5, with a power of 75% and alpha of 0.05. To increase the variation in the accelerometer data, we focused on participants involved in two broad types of work, including active and sedentary workers. Specifically, 15 health care workers recruited from the hospital—including physical and occupational therapists—were considered active, while 15 office staff—10 recruited from the hospital and 5 recruited from the university—were considered sedentary. Each participant provided written informed consent. The Academic Research Ethical Review Committee at Waseda University, Japan approved this study (2017-247).

### 2.2. Procedure

The participants were instructed to wear both a 350IT (old generation) and a 750C (new generation) accelerometer on the same belt (side by side) around the waist during all waking hours for seven consecutive days, removing the device only during water activities (e.g., bathing, swimming, and showering). The participants were also instructed to not modify their usual physical activity routine while wearing the devices. Using a seven-day diary, the participants recorded the time of day they started and ended wearing the device (the 350IT displays screen time), as well as making note if it was a work-day or a non-work day. For each participant, the positions of the devices worn on the left hip were randomized (i.e., anterior or posterior) [22]. Participants received in-person instructions on the wear and use of the devices. 

### 2.3. Accelerometer Devices and Data Management

The ASP 350-IT estimates metabolic equivalents (METs) every 10 or 60 seconds, based on the combined accelerations, measured by an internal tri-axial accelerometer (60-second epoch selected in this study). The criterion-related validity of the METs estimated by the 350IT was previously confirmed with the Douglas bag method [14]. The 350IT records anteroposterior (x-axis), mediolateral (y-axis), and vertical (z-axis) accelerations with a resolution of 3 mG at 32 Hz, and has the ability to classify physical activity into the locomotive and sedentary activities. The 350IT directly predicts the METs without the need of any additional process, using a multiple regression model, which is based on 12 key activities (7 locomotive activities and 5 household activities). This proprietary algorithm is able to distinguish the locomotive and household activity by the process filtered and unfiltered acceleration data [22]. The 750C uses the same algorithm as the 350IT. To be eligible for analysis, the participants needed to wear the accelerometer for at least four days (including one non-work day), with at least 10 h/day of wear time each day [23]. Non-wear time was defined as at least 60 consecutive minutes of <0.9 METs, with an allowance for up to 2 min of some limited movement (≤1.0 METs) within these periods [23]. The daily average time spent in SB (≤1.5 METs), LPA (>1.5 to <3.0 METs), MPA (≥3.0 to <6.0 METs), vigorous physical activity (VPA, vigorous physical activity ≥6.0 METs), MVPA (≥3.0 METs) [4,24], and step counts were calculated.

### 2.4. Statistical Analysis

The differences in the participants’ characteristics and mean daily minutes of SB, LPA, MPA, VPA, and MVPA between active and sedentary workers were estimated using independent *t*-tests to investigate if there were differences between the work type (i.e., active vs sedentary) and physical activity. Using paired *t*-tests, two-way random intra-class correlation coefficients (ICC) (2.1), and the Bland–Altman analysis, the difference and agreement between the two devices were analyzed for total and for active and sedentary workers, respectively. The Bland–Altman analysis assessed the bias, level of agreement, and systematic error of each intensity time between the devices [25]. Consistent with previous research, the limits of agreement were set at ±1.96 standard deviation of the difference scores [26]. The proportional bias, which is the proportionality effect to the magnitude of the measurement, was evaluated by Pearson’s correlation coefficients. The statistical analyses were performed using IBM SPSS Statistics 22 software (IBM Japan Inc., Tokyo, Japan). The estimates with *p*-values < 0.05 were considered statistically significant.

## 3. Results

After excluding ineligible participants (*n* = 4) who had missing data from invalid wear time, a total of 14 active workers and 12 sedentary workers (24–62 years) were included in the analysis. Table 1 shows the participants’ characteristics. There were no significant differences between the active and sedentary workers in terms of height, weight, age, and body mass index (BMI). However, there were some significant differences between the active and sedentary workers for the mean physical activity intensities measured using the 350IT. The active workers had significantly more MVPA (mean difference: 21.7 min/day, *p* < 0.01, 95% confidence interval, CI: 1.0 to 42.5 min/day), LPA (mean difference: 72.5 min/day, *p* < 0.01, 95% CI: 22.7 to 122.3 min/day), and less SB (mean difference; −124.4 min/day, *p* < 0.01, 95% CI: −209.9 to −38.9 min/day) than sedentary workers. There was no significant difference in accelerometer wear time between the active and sedentary workers (*p* = 0.42).

Table 2 shows the mean physical activity measures between the 350IT and the 750C, in the total, active, and sedentary workers. In the total sample, there were no significant differences between the means of the accelerometer wear time, VPA, and step counts between two devices. However, significant differences (*p* < 0.05) between the two devices for the estimated MVPA, MPA, LPA, and SB were observed. Among the active workers, the 750C measured significantly (*p* < 0.05) less MVPA, MPA, and VPA, and more LPA time, compared with the 350IT. Among the sedentary workers, the 750C measured significantly (*p* < 0.05) higher LPA time and less SB time, compared with the 350IT. While there were no significant differences between the means of the accelerometer wear time and step counts between the two devices in total, active, and sedentary workers, there was a significant difference in the mean of LPA between the two devices in total, active, and sedentary workers. The ICCs for all activities, estimated by the 750C and the 350IT, were high (0.94 to 0.99, all *p* < 0.001). The 750C had a tendency to measure more time in LPA, and less time in MVPA and SB than the 350IT. The Bland–Altman analysis showed no systematic bias (Table 3).

## 4. Discussion

To our knowledge, this was the first study comparing physical activity and SB data collected by two generations of Omron accelerometers, the 350IT and the 750C, in a free-living environment. In the total sample, with the exception of VPA, there were significant differences in all physical activity intensities between the 350IT and the 750C. The 750C measured a mean of difference of 2.5 min for MVPA, 2.4 min for MPA, 9.2 min for LPA, and 6.5 min for SB higher than the 350IT. However, there was no significant difference between step counts. The difference in mean minutes per day undertook in different physical activity intensities limits the comparability of the activity outcomes assessed by these two generations of ASP devices. The differences in measured physical activity are consistent with previous studies comparing the old and new generation of ActiGraph, which is one of the most-commonly used research-grade devices in the world [27]. For example, a previous study found activity differences measured by the old ActiGraph device (7164) and the new ActiGraph device (GT3X) ranged from 2.9 minutes/day for MPA, to 25.6 minutes/day with SB, and 31.2 minutes/day with LPA [28]. Our study provides unique evidence for the comparison between the new and old generations of Omron accelerometer: The key finding is that these two generation accelerometer devices cannot be used interchangeability in free-living environment studies. The different outcomes measured by these devices may be influenced by the participants’ specific activities (lifestyle) and the amount of time spent in their activities in a day. Thus, the discrepancies of estimating the METs in some specific activities between the 350IT and the 750C makes it difficult to compare the results and also means the devices are not interchangeable.

Our study used a free-living environment, therefore, laboratory settings may provide different results. However, previous studies using an accelerometer in a mechanical shaker demonstrated that accelerometer output provided a valid prediction of physical activity intensity [29]. Another previous study confirmed the differences among multiple generations of ActiGraph devices under a free-living environment and compared to using mechanical support [30]. Therefore, it is necessary that further validation of the 750C accelerometer is conducted to provide confirmation of the robustness of our results.

This study found differences in MVPA, MPA, VPA, and SB between the 350IT and the 750C depended on the participants’ work type. Considering the biases caused by the different activity pattern, it is assumed that the accuracy of the measurements between the 350IT and the 750C may be affected by variations in a specific activity. However, the 750C measured significantly more LPA time than the 350IT, regardless of the work type. The differences in LPA show that the 750C may be more sensitive in detecting LPA. Assuming that accuracy of measuring is updated with some modifications centering around LPA, this change in LPA may be why the devices found that differences in SB and MVPA changed depending on the participant work style. For example, the 350IT device may classify the activity as SB or MVPA, whereas, the 750C device may classify the same activity as LPA [31]. For over a decade, accelerometer devices have been advancing in the assessment of free-living physical activity and SB, especially LPA (mainly lifestyle activities). Although accelerometer devices have high validity and reliability of the measurement of physical activity and SB in laboratory conditions, it is difficult to accurately measure irregular LPA activities such as household, sedentary, and standing posture activities in free-living environments. Omron algorithms can classify activity into three different types (sedentary, household, and locomotive activities) [14,22]. Sedentary activity is measured by a lower acceleration signal than other activities. Therefore, it is easy for the 750C to include some acceleration noise outside of natural human activities and may misclassify some specific sedentary activities into the LPA category. Additionally, since sedentary workers accumulate more sedentary activities, we found a significant difference in assessed SB between these two devices compared to active workers. We also found differences among active workers with the 750C, which measured significantly less MVPA time than the 350IT. It is possible that some MVPA activities measured by the 350IT were misclassified as LPA, as the Omron algorithm has a tendency to underestimate the non-locomotive activities in the MVPA category [32]. One previous study has shown that 350IT-measured METs underestimated the postural activities and standing activities using the arm movement, compared with indirect calorimetric measurements as a criteria method [32]. Therefore, it also may be plausible that the measurable range of LPA in the 750C may be expanded in the direction of MVPA. These results among sedentary and active workers revealed the discrepancy in differences between the 350IT and the 750C may be due to the 750C being more sensitive to LPA.

This study had some limitations. Similar to other accelerometer comparison studies [30,33], this study had a small sample size. Since the participants in this study only included two specific types of full-time workers (sedentary and active workers), the results cannot be generalized to all full-time workers. Future studies need to use a deliberate selection of sedentary and active workers to increase variability in captured physical activity. Moreover, this study did not include a criterion measure of physical activity, therefore, inferences regarding device accuracy cannot be drawn from this study. This study had a power of 75% compared to the standard power of 80%. However, compared to previous studies that had a less or similar number of participants [34,35], we still found significant differences between the two devices. Therefore, being underpowered may not significantly impact our results.

## 5. Conclusions

This is the first study comparing the outcomes of two Omron triaxial accelerometer generations in free-living environments. The new generation device may not produce comparable physical activity outputs compared with the old device: The former may be more sensitive to LPA. The comparability of two Omron triaxial accelerometer generations is affected by the different types of physical activity. Exercise science and public health researchers and practitioners need to take into account these differences when using these accelerometer devices.

## Figures and Tables

**Table 1:** Participants’ characteristics.

Variable	Total	Active Workers	Sedentary Workers
*n* (men)	26 (16)	14 (8)	12 (8)
Height, cm	166.4 ± 8.0	166.9 ± 8.4	165.8 ± 7.7
Weight, kg	61.4 ± 11.9	62.9 ± 12.8	59.7 ± 11.0
Age, year	42.7 ± 8.8	40.1 ± 8.5	45.8 ± 8.6
BMI, kg/m²	22.1 ± 3.4	22.5 ± 3.8	21.6 ± 2.8

Note: Values are expressed as a mean ± standard deviation; There were no significant differences between active and sedentary workers; BMI = body mass index.

**Table 2:** Mean of accelerometer outcomes between 750C and 350IT devices.

Accelerometer Outcomes	350IT	750C	Difference (95% CI)	*p*	ICC (95% CI)
Total					
Wearing time (min/day)	834.0 ± 93.4	834.2 ± 92.0	−0.2 (−3.1, 2.6)	0.88	0.997 (0.990, 0.999) **
SB (min/day)	457.0 ± 121.0	450.5 ± 122.6	6.5 (1.4, 11.5)	<0.05	0.994 (0.981, 0.998) **
LPA (min/day)	303.8 ± 70.5	313.0 ± 71.3	−9.2 (−13.5, −4.8)	<0.01	0.983 (0.834, 0.996) **
MPA (min/day)	71.8 ± 26.9	69.4 ± 26.2	2.4 (0.9, 3.8)	<0.01	0.970 (0.790, 0.992) **
VPA (min/day)	1.4 ± 1.9	1.3 ± 1.8	0.1 (−0.1, 0.3)	0.20	0.974 (0.901, 0.992) **
MVPA (min/day)	73.2 ± 27.4	70.7 ± 26.6	2.5 (1.0, 4.0)	<0.01	0.969 (0.746, 0.992) **
Step counts (steps/day)	9127.2 ± 2903.4	9192.9 ± 2818.8	−65.7 (−176.5, 45.1)	0.23	0.993 (0.980, 0.998) **
Active workers					
Wearing time (min/day)	820.1 ± 88.2	822.4 ± 87.0	−2.3 (−6.3, 1.7)	0.24	0.998 (0.992, 0.999) **
SB (min/day)	399.6 ± 87.8	396.5 ± 89.0	3.1 (−2.5, 8.7)	0.26	0.991 (0.950, 0.998) **
LPA (min/day)	337.3 ± 69.6	346.7 ± 68.6	−9.3 (−14.7, −3.9)	<0.01	0.958 (0.806, 0.989) **
MPA (min/day)	81.1 ± 22.1	77.4 ± 21.6	3.7 (1.3, 6.1)	<0.01	0.996 (0.988, 0.999) **
VPA (min/day)	2.1 ± 2.3	1.8 ± 2.2	0.3 (0, 0.5)	<0.05	0.944 (0.826, 0.983) **
MVPA (min/day)	83.2 ± 22.3	79.2 ± 21.9	4.0 (1.6, 6.3)	<0.01	0.996 (0.988, 0.999) **
Step counts (steps/day)	10,079.7 ± 2585.3	10128.6± 2474.1	−48.9 (−222.6, 124.8)	0.55	0.996 (0.987, 0.999) **
Sedentary workers					
Wearing time (min/day)	850.2 ± 100.6	848.0 ± 99.5	2.2 (−2.0, 6.5)	0.27	0.998 (0.992, 0.999) **
SB (min/day)	524.0 ± 122.9	513.5 ± 129.5	10.5 (1.2, 19.7)	<0.05	0.991 (0.950, 0.998) **
LPA (min/day)	264.8 ± 49.9	273.7 ± 53.6	−8.9 (−17.1, −0.8)	<0.05	0.958 (0.806, 0.989) **
MPA (min/day)	60.9 ± 28.8	60.1 ± 28.9	0.8 (−0.7, 2.4)	0.27	0.996 (0.988, 0.999) **
VPA (min/day)	0.5 ± 0.9	0.6 ± 1.0	−0.1 (−0.3, 0.1)	0.37	0.944 (0.826, 0.983) **
MVPA (min/day)	61.5 ± 28.9	60.7 ± 29.0	0.7 (−0.8, 2.3)	0.32	0.996 (0.988, 0.999) **
Step counts (steps/day)	8015.9 ± 2959.4	8101.2 ± 2900.1	−85.3 (−245.2, 74.5)	0.27	0.996 (0.987, 0.999) **

**p < 0.001. Note: CI = confidence interval, ICC = intra-class correlation coefficients, SB = sedentary behavior, LPA = light physical activity, MPA = moderate physical activity, VPA = vigorous physical activity, MVPA = moderate-to-vigorous physical activity. Values are expressed as the mean ± standard deviation.

**Table 3:** Mean of difference and limits of agreement between 750C and 350IT devices.

Accelerometer Outcomes	Mean Difference	Limits of Agreement		*r*	*p*
		Lower	Upper		
Total					
SB (min/day)	6.5	19.2	−6.2	−0.13	0.53
LPA (min/day)	−9.2	−27.2	8.8	−0.07	0.72
MPA (min/day)	2.4	7.1	−2.3	0.19	0.35
VPA (min/day)	0.1	0.3	−0.1	0.35	0.08
MVPA (min/day)	2.5	7.4	−2.4	0.19	0.34
Step counts (steps/day)	−65.7	−194.5	63.1	0.31	0.12
Active workers					
SB (min/day)	3.1	9.2	−3.0	−0.12	0.68
LPA (min/day)	−9.3	27.5	8.9	0.10	0.72
MPA (min/day)	3.7	11.0	−3.6	0.13	0.66
VPA (min/day)	0.3	0.9	−0.3	0.37	0.20
MVPA (min/day)	4.0	11.8	−3.8	0.10	0.74
Step counts (steps/day)	−48.9	−144.7	46.9	0.37	0.19
Sedentary workers					
SB (min/day)	10.5	31.1	−10.1	−0.46	0.13
LPA (min/day)	−8.9	−26.3	8.5	−0.29	0.36
MPA (min/day)	0.8	2.4	−0.8	−0.04	0.90
VPA (min/day)	−0.1	−0.3	0.1	−0.36	0.25
MVPA (min/day)	0.7	2.1	−0.7	−0.06	0.86
Step counts (steps/day)	−85.3	−252.5	81.9	0.24	0.46

Note: SB = sedentary behavior, LPA = light physical activity, MPA = moderate physical activity, VPA = vigorous physical activity, MVPA = moderate-to-vigorous physical activity, r = Pearson’s correlation coefficient. Proportional bias indicated in statistical analysis. The mean difference was calculated as follows: 350IT-750C, the upper limits of the agreement were calculated as follows: mean difference + 1.96 standard deviation of the different scores. The lower limits of the agreement were calculated as follows: mean difference – 1.96 standard deviation of the different scores.
